# Targeted supplementation with bioactive plants sustainably improves goat health and decreases antiparasitic drug use on smallholder farms

**DOI:** 10.1038/s41598-025-34862-y

**Published:** 2026-03-03

**Authors:** Honest Machekano, Javier Ventura-Cordero, Paul M. Airs, Lovemore C. Gwiriri, Andrew Cooke, Joseph Virgil, Andrews Safalaoh, Patson C. Nalivata, Michael R. F. Lee, Taro Takahashi, Jan van Wyk, Eric Morgan, Casper Nyamukondiwa

**Affiliations:** 1 Department of Biological Sciences and Biotechnology, Botswana International University of Science and Technology, Palapye, Botswana; 2 Department of Zoology and Entomology, University of Pretoria, Private Bag X20, Hatfield, 0028 Pretoria South Africa; 3 Forestry and Agricultural Biotechnology Institute, University of Pretoria, Private Bag X20, Hatfield, 0028 Pretoria South Africa; 4 School of Biological Sciences, Queen’s University Belfast, Belfast, Antrim, UK; 5 Net Zero and Resilient Agriculture, Rothamsted Research, Okehampton, Devon UK; 6 Bristol Veterinary School, University of Bristol, Langford, Somerset, UK; 7 Animal Science Department, Lilongwe University of Agriculture and Natural Resources, Lilongwe, Malawi; 8 School of Sustainable Food and Farming, Harper Adams University, Newport, Shropshire UK; 9 Department of Veterinary Tropical Diseases, University of Pretoria, Pretoria, South Africa; 10 Centre for Agroecology, Water and Resilience, Coventry University, Coventry, UK; 11 School of Life Sciences, University of Lincoln, Lincoln, UK; 12 Department of Zoology and Entomology, Rhodes University, Makhanda, 6140 South Africa; 13 Centre for Environmental Policy, Imperial College London, Silwood Park Campus, Ascot, SL5 7PY UK

**Keywords:** Anthelminthics, Five point check, Nutraceutical plants, Plant-parasite treatment, Animal behaviour, Animal physiology, Ecology

## Abstract

Goats play a significant role in farming communities in semi-arid tropical areas with limited cropping capacity; however, production is limited by endoparasites, especially gastrointestinal nematodes (GINs). Control of GINs is typically mediated by anthelmintic drugs, but can be costly or ineffective where anthelmintic-resistant GINs are present. To manage anthelmintic resistance and improve herd health at lower costs, farmers can implement Targeted Selective Treatment (TST) strategies to treat animals based on performance or health traits. The study aimed to quantify the impacts of plant-based interventions on goat health, nutrition, and parasite infections when applied in a targeted selective feeding regime under an arid environment. Here we trialled a farmer-led TST scheme using a worm diagnostic tool for sheep and goats based on checking five points on the animal body; nose (purulent discharge), eye (colour of the conjunctivae), jaw (subcutaneous pitting oedema), back (body scoring condition, and tail (mild to severe diarrhoea). This method, termed the Five Point Check or FAMACHA, was used to periodically measure goat health, with anthelmintic interventions provided only to individuals in poor condition. In addition to TST with anthelmintic, a plant-TST was trialled on 50% of the farms where goats in borderline or poor condition were supplemented with local bioactive plants (*Viscum rotundifolium* L. or *Terminalia sericea*). Plant-TST treatment significantly reduced worm burden (*P* < 0.001) from a mean of 485 EPG pre-treatment to 269 EPG post-treatment, with 75% of goats having a FEC of ≤ 400 EPG. Further, goats under plant-TST had significantly improved health outcomes (*p* < 0.001 FAMACHA scores) compared to TST-anthelmintic. Goats under plant-TST were 46.6% less likely to require any anthelmintic treatment. Plant- and anthelmintic-TST had a similar FEC reduction (55.5% and 52.5%, respectively). Plant-TST offers a low-resource means to sustainably manage GINs in in goats in semi-arid conditions.

**Supplementary Information:**

The online version contains supplementary material available at 10.1038/s41598-025-34862-y.

## Introduction

Goats have a well-recognised value to rural livelihoods, providing a livelihood buffer particularly for communities vulnerable to climate change impacts such as crop failures. Thus, for many African countries, goat production contributes to several benefits such as food security, income, safety nets, and social status ^1^. These benefits have become even more important given the vulnerability of food and income systems to climate shocks and natural biotic pressures ^2,3^. Indigenous goats are well adapted to the harsh conditions often faced in arid environments and can efficiently metabolise the little vegetation available ^1^. In Botswana, as in many other countries, goat production is limited by parasites and diseases (especially endoparasites) alongside other risks such as predation, theft, biotic stress, and droughts ^4^. The cost of anthelmintics (including availability) exacerbates the challenge and thus, remains a key limiting factor, particularly for poor rural households in tropical environments. While a number of diseases can impact goat health, gastrointestinal nematodes (GIN) are a major cause of production losses worldwide and are especially detrimental in tropical conditions ^5^. This is partly due to the existence of more pathogenic species found in goats across the tropics, including *Haemonchus contortus* (Rudolphi, 1803), as well as species from *Trichostrongylus*, *Oesophagostomum,* and *Strongyloides* genera ^6^. Infection by these parasites reduce dry matter intake and feed digestibility, cause anaemia, and compromises milk and meat production ^7,8^. In addition, GINs affect goats’ welfare and severe helminthiasis tends to change the feeding behaviour ^9^. We thus hypothesize that low-cost anthelmintic options that deliver multiple benefits to goat production in the tropics are critical to sustaining rural smallholder farmers. The potential application of bioactive plants to control GINs in smallholder goat production frames this study.

Current goat management is largely traditional with a subsistence-oriented production characterised by limited parasite diagnosis and control mechanisms. These traditional production systems are characterised by informal, low skilled labour force, a small number of animals, limited resources, and overreliance on grazing and browsing of natural pastures as the main source of feed (Cooke et al. 2024). The seasonal availability and quality of feed thus remains one of the key challenges to productivity and thus limits relative disease-resistance conferred by nutrition. This is because during dry weather conditions, lack of rainfall and low temperatures cause herbaceous plants to defoliate, thereby reducing the abundance, quality, and variety of livestock feed (Cooke et al. 2024). Some of the fodder crops grown in the Southern Africa region include grasses, legumes and cereal crops. Examples of fodder grasses include Rhodes grass (*Chloris gayana*), Napier grass (*Pennisetum purpureum*), Brachiaria spp and Guinea grass (*Panicum maximum*). Legume fodder crops include lucerne (*Medicago sativa*), Desmodium spp, cowpea (*Vigna unguiculata*) and lablab (*Lablab purpureus*).

Tools for parasite management exist, but the availability of professional veterinary or extension officers is limited, alongside poor access to antiparasitic medicines (anthelmintics) for resource-poor low-income farmers ^10,11^. Complementary parasite control strategies have been developed*,* including improvement of animal nutrition ^12^ and hence the immune system ^13^. For example, treatment with copper oxide wire particles ^14^, breeding for genetic resistance ^15^, feeding with nutraceutical forages ^16^, and application of Targeted Selective Treatments (TST) ^17^, based on Five Point Check and the FAMACHA scores ^17,18^. The administration of anthelmintic drugs using the TST system selects individual animals requiring GIN deworming treatment based on threshold health-based criteria, enabling more efficient, efficacious and cost-effective drug use ^18^

Various versions of TST have been implemented in cattle, sheep, and goats, with the aim of delaying the development of anthelmintic resistance, through maintenance of nematode populations *in refugia* ^19,20^. TST, which in practice entails anthelmintic treatment of only a proportion of livestock herd, rather than the whole herd, leaves sections of the parasite population untreated (*refugia*), therefore providing untreated susceptible alleles that can cross with drug-resistant genotypes, diluting their persistence in the population ^21^. Several studies across Africa ^22–24^ and elsewhere (D’Amico et al. 2025), indicate increasing resistance to anthelmintics such as eprinomectin and albendazole, owing to uncontrolled excessive use or underdosing of goats. In developing countries, these practices are prevalent due to the high cost or poor availability of anthelmintics, among other factors ^23^. These factors, linked to under-, over-dosing, and the varying levels of resistance identified in several studies indicate the need for herd-specific management interventions such as TST explored in this study. TST has been successfully applied in goats reared in African production systems, to improve health and performance with significantly reduced treatment costs when compared to the ‘conventional’ whole-herd anthelminthic treatments ^25^. Studies have investigated different indicators to identify individuals within herds most affected by GIN ^26,27^, including the FAMACHA anaemia score of 1–5 with a higher score indicating anaemia), milk production, body condition score (BCS), live weight gain, diarrhoea score, and faecal egg counts (FEC) ^19,28^.

Anthelmintics are generally considered the primary treatment when TST is applied in livestock production. The same principle, however, might be used to direct complementary interventions to the best effect, including bioactive plants. These are plants that contain specialised metabolites (PSM) such as condensed tannins, which reduce parasitic burdens, increase protein absorption in the small intestine, and so enhance animal health and nutrition in the face of parasite challenges ^29^. Many studies have been conducted on the benefits of different plant species on parasites and on small ruminant performance ^16,30^. However, the potential to sustainably use locally available plant resources as nutraceuticals in arid environments deployed using a TST approach remains unexplored.

Here, we further hypothesise that feeding bioactive plants may be efficacious against GINs and reduce their effects on goats, and further that these benefits can be harnessed by application of TST. For many arid ecosystems, such as Botswana, these bioactive plants are readily available within goat grazing radii. If efficacious, bioactive plant applications could reduce the use of anthelmintic drugs, saving treatment costs and reducing negative impacts on the environment and ecosystem services ^31^, as well as slowing the development of anthelmintic drug resistance ^21^. By combining TST with targeted selective feeding of plant-based interventions, the study aimed to quantify the cumulative impact of plant-based TST interventions on goat health, nutrition, and parasite infections, with possible implications for reduced drug use.

## Results

### TST regimes buffer against seasonal GIN burdens

**Fig. 1:**
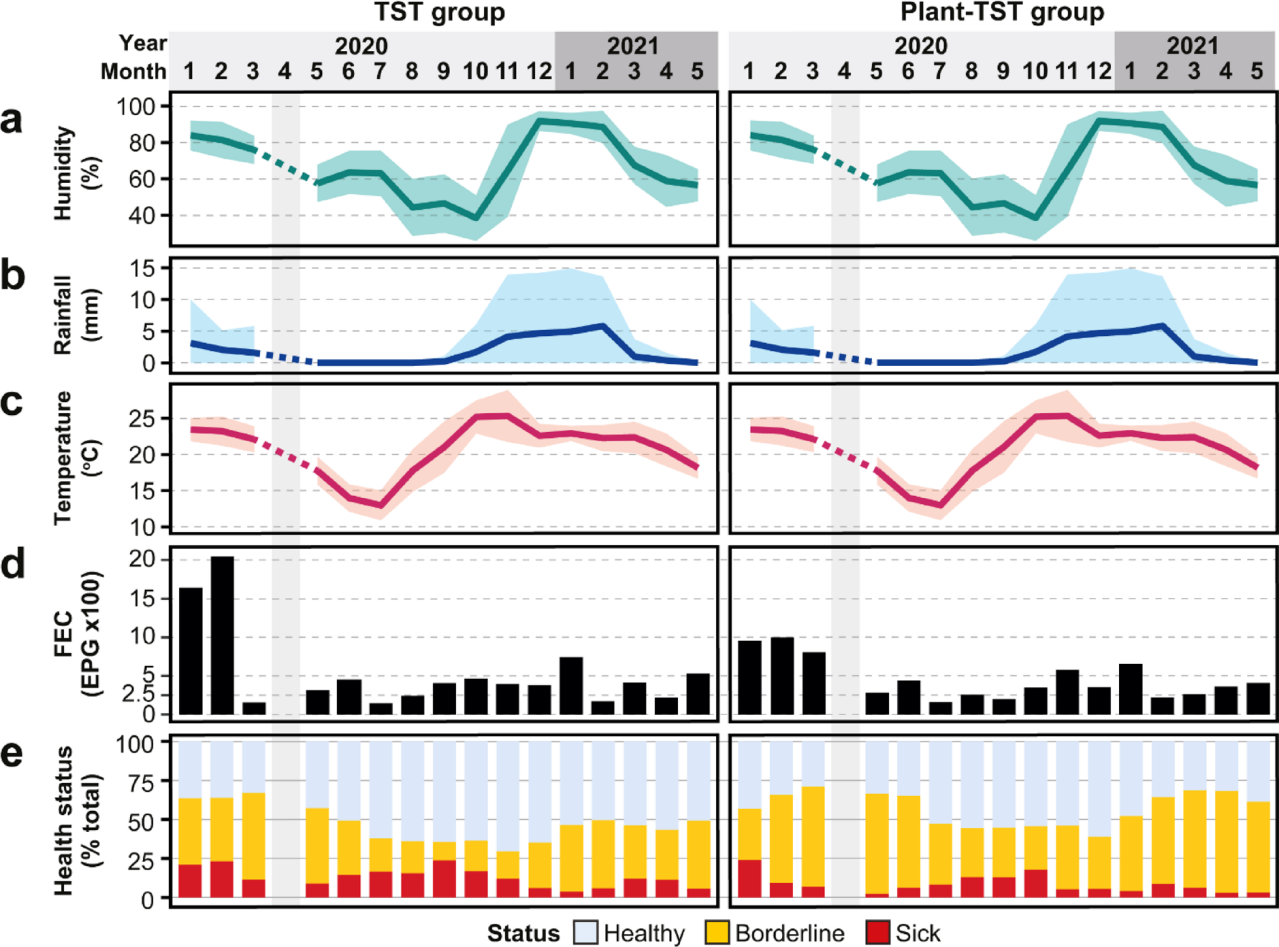
TST and Plant-TST regimes improve and maintain goat health regardless of seasonal parasite pressure. (**a**–**c**) Study area climactic conditions from NOAA/ESRL Physical Sciences Laboratory open data. (**d**) Monthly average strongyle nematode parasite abundance from study goats. FEC = faecal worm egg count, EPG = eggs per gram. (**e**) Monthly health status of all study goats. The month of the year is represented by a number under the year in the X-axis, where 1 = Jan up to 12 = Dec.

The highest precipitation, temperature and relative humidity were recorded during November-February in 2020-2021, corresponding to the main rainy season in the austral summer (Fig. 1a–c). Parasite egg output was similar between TST groups and tended to be highest in the summer (Oct-Feb); against this seasonal background, average EPG declined over the course of the experiment in both groups (Fig. 1d). The proportion of healthy goats in both groups increased during the rainy season, but was higher in the plant-TST group than the TST group (Fig. 1e).

Poor health was related mainly to high FAMACHA score associated with anaemia, which is often prevalent in goats with poor/low body condition score (i.e., emaciation), at different times of year. While FAMACHA and BCS are negatively correlated, FEC and FAMACHA were positively correlated. Body condition tended to be poor during the dry season, leading to peak anaemia prevalence early in the rainy season and persisted in the subsequent early rainy season (summer) months. Poor body condition and anaemia were more marked in the TST than the plant-TST group (Fig. S1). Bottle jaw and dag (diarrhoeal staining) were rarely observed and therefore made little contribution to the the overall health score, while nasal discharge was common, mostly in the dry season (see Fig. S1).

### Monitoring effect of TST regimes on smallholder goat health

**Fig. 2:**
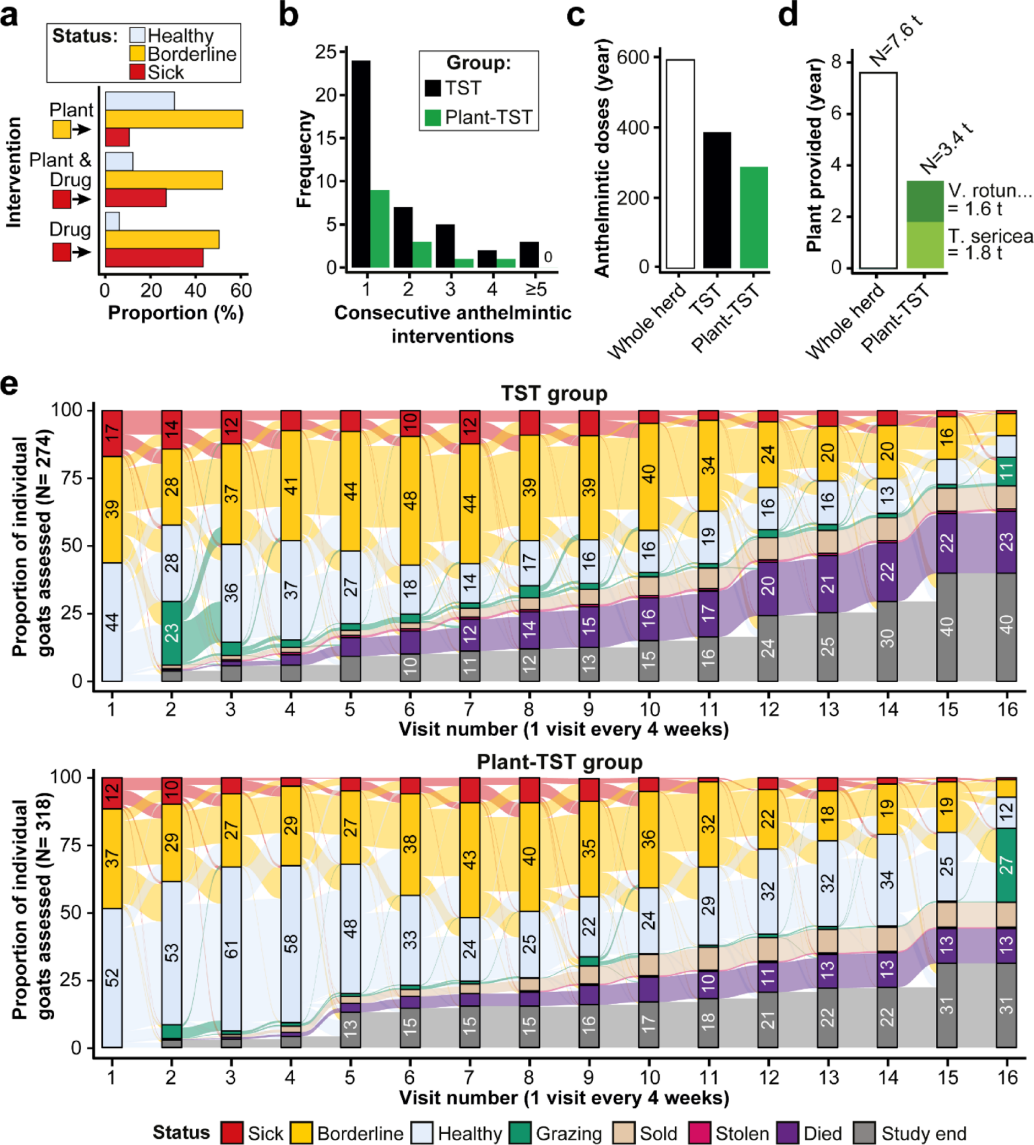
Tracking impact of TST and plant-TST regimes on goat health. (**a**) Goat health status changes after applying plant supplementation (borderline individuals), anthelmintic plus plants, or anthelmintic alone. (**b**) Consecutive anthelmintic interventions required for the same goats after an initial intervention. (**c**) Total anthelmintic interventions provided per year for TST and plant-TST regimes as compared to a traditional whole herd treatment regime. (**d**) Beneficial plant resources used per year for plant-TST regime goats as compared to a whole herd supplementation regime for the same herd size. (**e**) Proportion of goat statuses in TST and plant-TST regimes scaled by visitation per farm. Proportions ≥ 10 shown in bars. Grazing = goats out to graze when FPC was performed, study exit = visit number at the end of the study or when farmers left the study.

Following plant supplementation of goats at borderline health status, most animals remained in borderline health at the next evaluation, while more goats returned to good health than those that deteriorated (Fig. 2a). Of those goats in poor health and consequently receiving anthelmintic treatment, a significantly higher proportion improved in health status when offered bioactive plant supplementation (plant-TST group) than those given only anthelmintic drug (TST) (Chi-square test, df = 4, *P* = 0.0001, Fig. 2a). Offering an extra quantity of bioactive forage with anthelmintic intervention also reduced the number of goats requiring more than three anthelmintic interventions during the study (Fig. S2), and those needing repeated anthelmintic treatments across consecutive monitoring intervals to return to healthy status (Fig. 2b).

Overall, use of targeted interventions reduced the use of anthelmintic drugs in both TST groups (Fig. 2c). Plant supplementation in a targeted manner also reduced plant use by > 50% compared to a whole herd feeding strategy (Fig. 2d).

Over the whole study, proportionally fewer goats in the plant-TST group were sick or borderline, and healthier than in the TST group (Fig. 2e). Out of goats with borderline status in the plant-TST group (and were therefore supplemented with the experimental plants), 46.6% never required a drug intervention compared to 56.6% in the TST where drug intervention was used. Goats becoming sick in the plant-TST group more commonly transitioned back to borderline status rather than remaining sick, than in the TST group (Fig. 2e).

Kaplan–Meier analysis confirmed a significant difference in the need for drug intervention between TST and plant-TST groups (Chi-square, df = 1, *P* = 0.0007). Goats in the plant-TST group survived more time without entering the sick category and hence being treated; based on pooled data, the TST group reached the median, i.e., 50% of goats treated at least once, at day 244 (95% confidence interval 214–412 days), whereas in the plant-TST fewer than half the goats were treated before the end of the study (Fig. S3). Successive periods of bioactive plant supplementation prolonged the time until first treatment, and the most favourable survival curve was achieved with goats supplemented four or more times in plant-TST group (Fig. S3, Chi-square, df = 5, *P* = 0.001). Based on individual plant intervention, more than 80% of animals supplemented with *T. sericea* and *V. rotundifolium* when in borderline health status maintained acceptable health without anthelmintic drug intervention up to 228 and 204 days, respectively, with *Terminalia* having the stronger effect (Fig. S3, Chi-square, df = 2, *P* = 0.0002).

### Effect of plant-based intervention on parasite infection and goat performance

Most (75%) goats associated with a healthier outlook at the time of inspection showed a FEC of ≤ 400 EPG (range, 0–6050 EPG). Lactation status, FAMACHA, BCS, plant intervention, and the time points sampled were factors affecting FEC of goats in both TST groups. Non-lactating goats had lower FEC than lactating goats (*P* < 0.05, Table S1), and lactating goats tended to have a relatively lower BCS than non-lactating goats. There was a tendency towards higher FEC in paler goats, establishing a positive correlation between FEC and FAMACHA scores. Those goats showing high FAMACHA scores of 4 or more (i.e., more anaemic) displayed significantly higher FEC than goats with FAMACHA scores of 3 and below (Table S1, *P* < 0.05). BCS was consistently negatively correlated with parasitic burden, with FEC higher in goats with lower/poor BCS. For instance, goats with a BCS of 1.5 often had a high FEC > 500 EPG (Table S1); However, very emaciated goats (BCS 0.5) did not have higher FEC. On average, FEC were highest during the rainy season, exceeding 500 EPG from January to March and then decreasing through the dry season. Finally, goats supplemented with *T. sericea* showed a lower FEC than those fed with *V. rotundifolium* (Table S1, *P* < 0.05), with intermediate FEC on average in the TST group, however, this comparison does not consider the different levels of drug treatment between groups.

The extent to which the TST regimes affected goats’ BW was evaluated. BCS was negatively correlated with submandibular oedema, nasal discharge and dag score. Goats in better body condition (BCS > 2.5) were heavier than goats in poor body condition, while those with submandibular oedema or nasal discharge were lighter (lower BCS) than those without these conditions (Chi-square, df = 2, *P* = 0.001, Table S2). Goats with high dag scores also had low BW. There was no clear relationship between BW and FAMACHA score. Seasonally, BW was highest during and soon after the rainy season (Jan-July) than in the dry season, especially in the first year of study. Aggregating health data across the FPC, goats did not differ in BW across healthy, borderline or sick status, but goats in the plant-TST group were overall heavier than those in the TST group (Chi-square, df = 2, *P* = 0.006, Table S2).

The FEC decreased significantly in both groups following anthelmintic drug intervention (Fig. S4, W test, TST, *P* < 0.0001, plant-TST = 0.0001, respectively). The means for plant-TST were 485 EPG and 269 EPG for pre- and post-treatment, respectively. Similarly, TST displayed a mean of 365 in the pre-treatment and 192 in the post-treatment. The FEC reduction for each plant-TST and TST was 55.5 and 52.5%, respectively.

## Discussion

The study set out to determine the benefits of selective supplementary feeding with locally available bioactive plants on the health of goats in arid Botswana, to reduce the need for treatment with chemical anthelmintics for improved goat health, and lower production costs while simultaneously delivering sustainable environmental and socio-economic benefits. It was hypothesised that plant supplementation applied as plant-TST reduces GIN infections and improves health status through improving nutrient supply and/or through direct ‘pesticidal’ impacts on the parasites themselves. Aiming to reduce the demands on local plant resources, only those animals showing ‘borderline’ suboptimal health through a range of indicators were strategically supplemented with bioactive plants in a targeted selective manner.

### Effect of plant-based intervention on goat health

Following a deterioration of health to borderline status, bioactive plant supplementation succeeded in reducing treatment requirements. Goats benefitting from targeted plant supplementation remained in acceptable health for longer, deferring the need for anthelmintic drug intervention, and also required fewer repeated anthelmintic treatments to restore health, compared with drug-only (TST) intervention.

In agreement with the present results, Marume et al. ^32^ found that goats supplemented with *Acacia karro* showed better FAMACHA scores (low anaemia) than a control group without supplementation. Goats fed with tannin-rich *Lespedeza cuaneata* pellets maintained a FAMACHA score of 2 after 29 days of supplementation ^33^. Likewise, higher body condition was observed in Cashmere goats fed heather (*Calluna vulgaris*) from August to September ^34^ and Paolini et al. ^35^ reported that repeated supply of *Onobrychis viciifolia* was beneficial for dairy goats through reduced FEC, and improved resilience and resistance to GIN infection as also recently reported by Quadros & Burke ^36^. In the present study, there was a clear benefit of bioactive plant-feeding to goats in borderline health status (plant-TST). Direct and indirect effects of PSM against GIN in small ruminants have been documented, *i.e.,* low L3 establishment, lower fertility and fecundity in female worms, and impaired development from one life stage to the next ^29,37^. This could decrease worm burden, thus the development of anaemia, as deduced from better FAMACHA scores on the monitored farms with plant supplementation. Alternatively, or additionally, the extra protein and energy in the supplemented goats could be beneficial in improving resource availability, compensating for protein loss and enabling repair from parasite damage. While the studies highlighted above ^32–35^ explored the combined effects of nutrition and bioactive plant components, the current study further explored the intermittent application (TST) of bioactive plants on goat performance and health. Here, our study indicates that a combination of better nourishment and antiparasitic effects, applied strategically through TST, appears to positively affect (improve) goats’ health.

It is already appreciated that nutritional supplementation is a sound complementary management strategy for GIN infection in small ruminants ^12^. Nevertheless, as not explicitly outlined is previous studies, it is important to specify when and for how long extra feed should be offered. Contrary to previous studies based on artificial goat infection and indoor feeding regimes under strict researcher managed conditions ^38,39^, our study was based on natural worm infestation under free range, farmer managed conditions subjecting the treatments to the typical reality for target farmers and their livestock management conditions, making the results more applicable with potentially long-lasting implications. In addition, our study provides results for a year and half (17 months), a trial period long enough to validate the consistency of the observed trends, whereas most previous studies were either limited to animal data records for a few days ^38,40^ or a few weeks ^39,41^. Further to that, whereas most studies focussed on already known cultivated plants ^38,39,41^ that require cultivation or purchasing and applied on whole herd basis, our study focussed on naturally occurring indigenous bioactive plants minimally applied through the use of plant-based TST. The rationale is that if this is done on a continuous whole-herd basis without TST as in our case, resource inputs become substantial and local bioactive plant resources could also potentially become denuded over time. Based on the survival analyses presented here, anthelmintic treatment was deferred by a single plant intervention of 8–12 days’ duration within a month. The benefit of supplementation increased with subsequent rounds of feeding, such that four or more plant interventions significantly increased the survival probability without a deworming intervention. Consequently, a repetitive supplementation plan is necessary. Resource requirements are, however, much reduced if focused on the individual goats in need through borderline health status checkpoint, *i.e.,* a TST regimen. For example, feeding of ~ 250 g FB of fresh *T. sericea* and *V. rotundifolium* per day was sufficient to achieve the above benefits for goats in Botswana. Supplementation with *T. sericea* showed better survival curves than with *V. rotundifolium* or without targeted plant supplementation.

### Effect of health and plant-based intervention on goat performance

The FPC is helpful to rapidly determine signs of parasitism in small ruminants and to identify the individuals most in need of treatment ^42^. Since FPC indicates ill health, it might be expected to correlate also with body weight variation in adults. Our results partially confirm this hypothesis. For example, the presence of nasal discharge and submandibular oedema positively correlated with low BW compared to goats without those signs. Submandibular oedema is associated with chronic multiple GIN infections and nasal discharge with the presence of larvae of the nasal bot fly, *Oestrus ovis*, although goats seem to be less affected by this insect than sheep ^42,43^. On the other hand, there was no clear relationship between the Dag score and BW (Table S2).

Climatic conditions, mainly precipitation, drove fluctuations in goat BW: higher BW was recorded during the rainy season than the dry season. Hoste et al. ^12^, posed the question regarding the most appropriate timing during the year to offer a supplementary crude protein (CP) and energy to goats. Our study showed that BW was lowest between September and October. Thus, we propose that within the context of our results, this could be the optimal timing during which supplementary nutrition could have the greatest impact. Moleele et al. ^44^ published that *T. sericea* leaves, together with pod litter was the most valuable plant in the cattle diet (32% of the total diet) during September due to scarce nutrients at the end of the dry season in Botswana. Likewise, Madibela et al. ^45^ reported that goat smallholder farmers from Botswana fed with several parasitic plants, including the mistletoe, *V. rotundifolium*, during the dry season, when they remain in-leaf. Goats might find similar resources during grazing or farmers could implement the “cut and carry” system suggested in the present study. The frequency with which bioactive trees and other plants can be harvested is another consideration that affects implementation and sustainability. Moyo et al. ^46^ reported that to guarantee sustainable use of *T. sericea,* trees needed at least three months to restore their reserves after several harvesting events, but they also recommended rotational periods of at least six months after the first cut. Moreover, it is necessary to design a strategy together with goat smallholder farmers to consider the number of trees to be alternately harvested sustainably over the seasons, to safeguard against overexploitation.

The positive impact of applying plant supplementation in combination with TST (plant-TST) on goats’ health was demonstrated and extends to impacts on performance, as indicated by BW. Goats classified as sick (FAMACHA 4 and 5), borderline (FAMACHA 3) and healthy (FAMACHA 1 and 2) were heavier in plant-TST than their counterparts in the TST (drug only) group, which could be related to improved nutrition of these animals, a lower GIN burden, or a combination of both. Improved nutrition was reported to improve the host animal’s resistance to gastrointestinal nematodes through nutrient partitioning. Abundant nutrition reduces the need for prioritization of nutrients to immunity acquisition, but also ensures that there is enough nutritional resources for immunity responses to draw from ^47,48^. Most of the indigenous savanna plants contains alkaloids, condensed tannins and flavonoids ^49^, which were reported to have anthelminthic effects ^50–52^. In addition, maintenance of high protein buffers the animal from parasite induced leakage of nutrients, particularly plasma proteins guaranteeing animal survival ^47,48^.

As reported by smallholders, the main diet of the goats is based on shrubs, grasses and some crop residues during dry seasons. The most common grasses consumed by goats are in common grazing or ‘Dambo’ areas, and these grasses are characterised by a low CP content ^53^. In contrast, the experimental plants offered during the study trial displayed a medium to high CP content (> 7%) and low fibre components (see Table 1). The results are consistent with a previous study where goats supplemented using Acacias and *Leucaena leucocephala* showed better BW gains than non-supplemented goats, which was attributed to high CP, low fibre content and high digestibility ^54^. Both *T. sericea* and *V. rotundifolium* increasing the BW of strategically supplemented goats; heavier animals could increase farmers’ profit and the resilience of goats to adverse events including drought, encouraging farmers to adopt this management strategy as a regular practice in their production systems.

**Table 1:** Proximate feed analyses based on the plants collected by farmers for nutritional supplementation during the wet season.

Plant species	ID	DM ± SD		OM ± SD		Ash ± SD		CP ± SD		NDF ± SD		ADF ± SD		CT ± SD^†^		AH
*Viscum rotundifolium*	PC1	47.0	4.05	93.1	1.70	6.9	1.71	25.8	2.32	30.0	2.07	21.1	1.65	4.9	1.92	^68^(Tibe et al., 2013)
	PC2	46.7	4.86	92.8	0.96	7.2	0.95	25.1	2.34	23.9	4.17	18.8	1.10			
	PC5	50.0	3.75	93.1	1.65	6.9	1.62	23.9	1.10	29.8	3.61	21.3	1.42			
	PC6	51.3	3.32	92.7	0.70	7.4	0.70	19.9	4.99	21.6	3.00	18.7	2.75			
	PC7	47.5	1.96	93.5	0.95	6.4	0.95	22.9	2.02	24.6	3.68	20.7	3.73			
*Terminalia sericea*	PC3	52.3	3.61	96.2	0.07	3.8	0.06	10.2	0.85	46.8	4.34	36.4	6.39	3.0	0.05	^67^(Aganga et al., 2006)
	PC8	52.6	2.57	95.4	0.59	4.6	0.58	10.8	2.93	41.8	7.23	33.0	5.89			
	PC9	46.5	1.13	96.1	0.85	4.0	0.85	7.7	2.76	42.2	0.60	31.6	3.34			
	PC10	50.4		95.5		4.5		8.8		23.2		15.7				
	PC11	52.1	0.64	96.2	0.85	3.8	0.86	10.3	1.05	33.1	7.28	26.9	9.75			

DM, dry matter; OM, organic matter, CP, crude protein; NDF, neutral fibre detergent; ADF, acid detergent fibre; ^†^CT, average condensed tannin equivalent to leucocyanidins; AH, Anthelmintic activity published previously.

### Effect of plant-based intervention on parasite infection

Estimations of the parasitic load based on GIN egg output (FEC) has been studied as a criterion for treatment when TST is applied in goat farms ^27,55^. However, this requires repeated sampling by farmers, including access to equipment, training and a swift, but affordable diagnostic service. Health indicators such as the FPC are arguably more practical but are not direct indicators of parasite burden. In the present study, only two components of the FPC affected FEC, namely FAMACHA and BCS. It has been reported that high FAMACHA scores are directly related to anaemia due to high *H. contortus* burden in small ruminants ^42^. Goats in the present study showing FAMACHA score of 4 had higher EPG than those with a FAMACHA score of 3.

There was also a difference in the FEC of goats with a BCS of 2 vs 1.5, which could be explained by better body reserves conferring protection from GIN, or by parasite burden denuding body reserves. Goats with low BCS would require more anthelmintic interventions under FPC-based TST, and this would lead to a disproportionate impact on egg output at group level. Mahieu et al. ^56^ described poor grazing conditions causing low BCS, low kidding rates and increased deworming needs in Creole goats. BCS is a practical tool that with practice and training, is a good criterion for the purposes of TST ^55^.

It is well recognised that the relaxation of the immune system responses during the pregnancy in small ruminants increases GIN burden, through peri-parturient rise in the FEC. A significant difference in the parasite burden between lactating and non-lactating goats was recorded in the present study, although no difference was observed in pregnant does. This was also noted by Malan et al. ^57^ who reported that of dry, pregnant and lactating sheep ewes that grazed as a single flock on pasture under conditions of severe *H. contortus* challenge, respectively 17%, 29% and 55% required anthelmintic treatment under a TST regime. The lactating period is highly demanding nutritionally, and lactating goats therefore have fewer reserves to face parasite infections. The findings in this study agree with a study performed in Creole goats infected by mixed natural GIN, in which reduced FEC were observed in does after cessation of lactation ^58^.

Climatic conditions partially explained and contributed to the FEC pattern in both TST experimental groups, as discussed above. However, there was no evidence to identify any effect on parasite burdens from TST regime (plant + drug or drug-only), health status or its interaction. The first three months of the study (January to March 2020) recorded higher FEC than the corresponding period in 2021, even though higher precipitation during the latter period would be expected to favour parasite transmission. This might suggest that parasite burden declined over time due to the sustained application of TST. If true, this would indicate epidemiological benefits from TST, reducing onward infection pressure and elevating the health of goats.

A parasitological comparison was also performed within the plant-TST group, in terms of which plant had most effect on FEC. Goats fed with *V. rotundifolium* had higher FECs than those supplemented with *T. sericea*. Several negative effects of PSM on GIN have been reported, which could explain lower FEC in supplemented goats ^29^. However, *T. sericea* and *V. rotundifolium* expressed a similar quantity of condensed tannin as 3.0 vs 4.9% leucocyanidin, respectively. It is also possible that goats supplemented in the kraals as part of TST ingested less forage than those that were grazing normally, and thus ingested fewer parasites, a phenomenon named the substitution effect ^37^.

The effect of anthelmintic treatment was also evaluated in both TST experimental groups. A significant reduction in FEC was observed in the goats treated, but it was observed in some cases that FEC did not reduce and even increased after drug intervention. Similar results were recently found in one out of 10 goat smallholder farmers from three villages in Gaborone, Botswana, where faecal egg reduction tests were performed using ivermectin anthelmintic ^59^ and suggested the presence of drug resistance. While this study was not designed to evaluate anthelmintic resistance, it is essential to carry out more studies to investigate the extent of this problem among resource-poor farms in Botswana and beyond. By training local farmers in the application of TST and the additional insertion of plant-based interventions into this approach, selection pressure for anthelmintic resistance should be reduced relative to whole-herd treatments ^25^.

Impacts of TST on parasite populations will interact with those of climate and weather ^60,61^. (Santos et al. ^62^ concluded that temperatures of 19–42 °C, > 68% relative humidity (RH) and measurable precipitation favoured GIN migration onto herbage, especially for *H. contortus*. The present results showed similar trends, where FEC followed a clear tendency corresponding with the precipitation, temperature, and humidity during two rainy seasons, peaking from February to March 2020 and October 2020 to January 2021. A similar pattern was observed in small ruminants from Nigeria, where GIN prevalence and FEC were paralleled by rainfall pattern showing a seasonal sequence ^6^.

Goat-keeping smallholder farmers in Botswana and other arid tropics should be particularly vigilant for signs of GIN infection during these critical months and consider interventions including those evaluated in the present study. Conversely, during the dry season, when GIN infection appears to be low, farmers should emphasise the enhancement of goat nutrition by supplementing with local plants of high nutritional value to improve animal anthelminthic resilience and survival through nutritional benefits ^47,48^. The latter has been reported to improve goats’ resilience in tropical conditions independently of worm burdens ^63^. The implementation of specific plant-based supplements targeting individual goats on the basis of established health indicators has the potential to dynamically meet the twin challenges of poor nutrition and parasite infection, utilising local resources in a sustainable and pragmatic way.

The value of plant-TST is to prevent long-term socio-economic losses associated with parasite-related goat mortality. Krecek and Waller ^5^ found out that annual losses to farmers associated with parasites were estimated to be US$26 – 45 million in Kenya and South Africa. Further, prophylactic anthelmintic administration and routine treatment contribute to high drug costs for smallholder farmers, which undermines the profitability of smallholder farmers who often have small herds ^32^. The plant-TST thus, enables resource-poor farmers to mitigate parasite-related mortality. More recently, the drive towards organic chemical-free meat, as well as public concern associated with the risk of residual chemical accumulation in milk, meat and meat products, underpins the wider potential long-term benefits of plant-TST ^12,64,65^.

## Conclusions

A positive effect of TST on goat health was observed, which was enhanced with specific plant supplementation (plant-TST) based on goat health indicators. Plant supplementation significantly increased the proportion of goats in a healthy status under plant-TST and reduced the need for anthelmintic drug treatment. On average, goats supplemented with *T. sericea* and *V. rotundifolium* lasted longer without the need for anthelmintic drug intervention than non-supplemented goats. It was determined that TST with plant supplementation positively affected the goats’ nutrition, leading to healthier goats in the plant-TST than the TST group. FPC scores and sampling time influenced the goats’ BW. Nevertheless, it was not possible to identify an effect on GIN burdens by TST group, health status or its interaction. Further research could group the goats by age and/or sex, and repeat the trials including mixing the two plants under controlled conditions. In addition, *in-vitro* experiments with metabolites from *T. sericea* and *V. rotundifolium* could also provide further insights. Precipitation, temperature, and humidity drove seasonal FEC patterns during the experimental study; while FAMACHA, BCS, lactation status, sampling time and plant intervention affected the observed FEC. Goats fed with *V. rotundifolium* showed a higher FEC than *T. sericea*. Therefore, TST incorporating locally available bioactive plants could be used as an efficacious and sustainable method for GIN control in arid smallholder resource-poor farmer environments in developing countries. Furthermore, by being used in combination with anthelmintic drugs, this approach can save costs associated with anthelmintic drugs, delay the need for anthelmintic drug intervention and hence reduce drug resistance and ecological disservices associated with anthelmintic drug use, while simultaneously improving productivity. Use of plant-TST for parasite control may be the first step towards the sustainable and integrated management of helminth parasites compatible with low-resource farmers in arid farming environments. The study showed that, practically, farmers can use the five-point check to implement plant-TST based on locally occurring bioactive plants, resulting in sustainable management of GINs.

## Material and methods

### Study area and farmer recruitment

The study was carried out from January 2020 to May 2021 (17 months) in the Central District of Botswana, an area considered predominantly semi-arid ^66^. Mean annual rainfall in Botswana is spatially and temporally variable, ranging from 250 mm in the south-west to 650 mm in the north-east and mean minimum and maximum daily temperatures ranging from 5–22 ⁰C in peak winter to 19–33 ⁰C in summer ^66^. The study area has a mean annual temperature of 28.5 ºC ^66^ and average annual precipitation of 400 mm ^67^. Humidity, precipitation and temperature information were provided by the NOAA/ESRL Physical Sciences Laboratory for the duration of the study (http://psl.noaa.gov/).

**Fig. 3:**
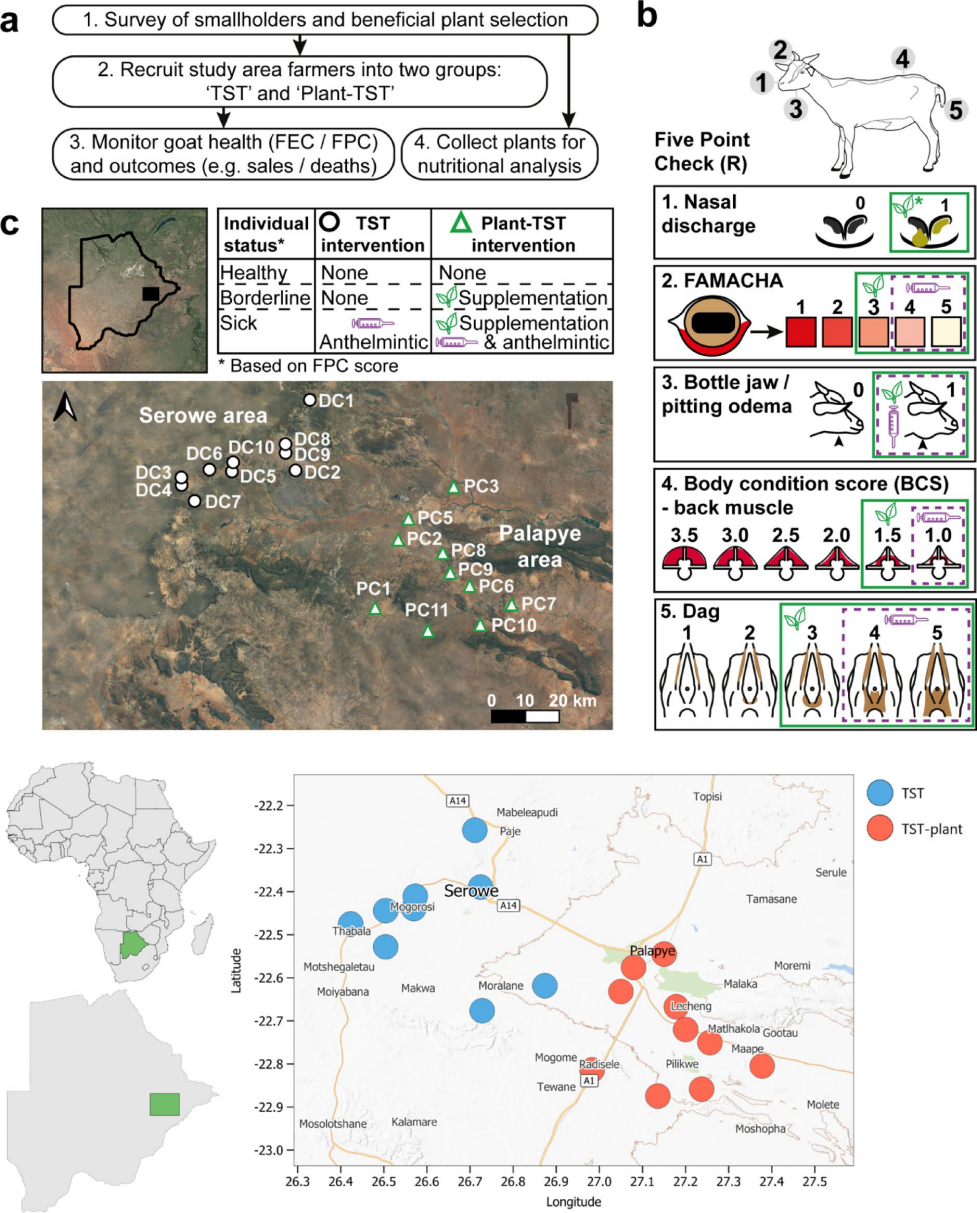
Overview of the targeted selective treatment program (TST) with plant supplementation. (**a**) Experimental design to assess the impact of TST across two groups, one with anthelmintic intervention only (TST) and one with plant supplementation in addition to anthelmintics (Plant-TST). FEC = faecal worm egg count, FPC = Five Point Check. (**b**) Five Point Check treatment decision chart with thresholds set for TST interventions with plant supplementation (leaf symbol) and anthelmintics (syringe symbol). (**c**) Overview of study area. (**d**) map of the study sites. TST intervention groups (PC = Plant-TST group, DC = TST group. Maps were created by authors using open source QGiS 3.18 version ^70^ (Fig. 3c) and QGIS 3.44.5 (2025) (Fig. 3d) (OpenSource Geospatial Foundation Project. (http://qgis.osgeo.org).

A pre-project survey of 63 goat farmers from greater Palapye and Serowe was carried out through face-to-face questionnaire interviews in September 2020. This aimed to gather information on goat management and health and possible bioactive plant candidates for feed supplementation based on indigenous knowledge (Fig. 3a). A snowball sampling method was employed to recruit the farmers for the survey. With veterinary extension personnel support and guidance, the pre-survey data was used to pre-select experimental farms/farmers, based on the number of goats the farmer had (15 – 50 goats), age of the farmer, kraal accessibility by a vehicle, goat keeping experience (at least 3 years) and willingness to participate. All procedures involving farmers and collection of their data were done in accordance with the ethical standards and guidelines approved by the Biosafety and Research Ethics Committee of the Botswana International University of Science and Technology, research permit reference # ENT8/36/4 XXXX II (5), provided by the Republic of Botswana. All participating farmers were fully informed about the study objectives and procedures, and provided their voluntary, written informed consent prior to participation. This resulted in ten farmers of mixed ages with a 7:3 and 4:6 female to male ratio in the TST and plant-TST, respectively, resulting in an overall 55% female and 45% male farmer participants. The most frequently mentioned plants were further screened based on (i) previous reports on anthelminthic effects in literature, mammalian toxicity, local and historical reports of use on goats, and their availability and abundance (plus availability) in target communities. The second screening resulted in the selection of two species for this study, (i) a mistletoe, *Viscum rotundifolium* L. (Santalales: Santalaceae) and a deciduous tree, *Terminalia sericea* (Burch) (Myrtales: Combretaceae) ^68,69^. These are plants that the farmers were already using to feed their goats on a ‘cut and carry’ basis, culturally, but without the scientific knowledge of the benefits. The plants naturally occur in the farmers’ grazing areas. As this was a participatory study, there was no need for approval or ethical clearance regarding the farmers collection of the plants to the kraals for feeding the goats. This practice is part of the farmers’ continuing culture, the role of the researchers was to measure quantities and guide on controlled feeding (frequency) of the target goats. Plants were sourced within the farmers’ grazing areas, within the radius of greater Palapye village in Botswana, the nearest point: S22° 34′ 32.6″; E27° 04′ 47.0″, and furthest point: S22° 59′ 29.8″; E27°17′ 16.9″. Table 1 shows the chemical analyses from both plant species and the PSM contents. Signed individual consent forms to participate in the project and approval for their goats to be used in the study were sought from each of the selected farmers before the project commenced. The willing farmers were then split into two groups by area (location) to avoid cross-contamination between the main parasite interventions. The signed consent forms are attached. TST group farmers (DC code) and Plant-TST (Code PC) group, making a total of 11 villages across the two groups. For each group of selected farmers, a meeting was arranged to explain methodology, processes, goat health monitoring by FPC, and the TST concept (Fig. 3a, b, c, d).

Six Hygrochron iButtons (Dallas Semiconductors) (0.5 °C and 0.5% temperature and relative humidity accuracy, respectively; 1 h sampling frequency) were placed about 2 m above ground, fixed on a wooden pole/tree in the kraal on three of each Plant-TST and TST farms, to monitor relative humidity and temperatures at the study sites and to complement centrally accessed data.

### Health monitoring and targeted selective treatment regimens

At the start of the study and during subsequent visits, researchers inspected goats and implemented the FPC for health indicators (Fig. 1b) ^42^As the study progressed, farmers were trained to apply the checks themselves, and agreement with the researcher’s scores was assessed for validation. The FPC system was developed to identify the negative effects of GIN infections in small ruminants. The BCS was adapted according to the scoring system for small African goat breeds published by Honhold et al. ^71^, on a four-point scale including half-score intervals. All experimental protocols in the methods were carried out in accordance with the ethical standards and guidelines approved by the Biosafety and Research Ethics Committee, Botswana International University of Science and Technology, research permit reference # ENT8/36/4 XXXX II (5), provided by the Republic of Botswana. In addition, all experiments were carried out in accordance with ARRIVE international guidelines.

Using pre-survey data (n = 63), two experimental plant species were selected for evaluating their effect on parasite infection, health, and nutrition in goats. *Terminalia sericea* and *V. rotundifolium* were chosen as plants that are locally available, abundant, already fed to goats, and nontoxic; as well as reported potential for anthelmintic activity ^68,69^.

The goats’ basal diets and grazing times were determined from interviews with each farmer. Experimental plants and basal diet samples were collected at 4-week intervals to evaluate their nutritional value. From each feed, approximately 300 g fresh basis (FB) was collected and dried in an oven at 60 °C for > 48 h until reaching constant weight and then stored in labelled oven-compatible pockets prior to laboratory analysis.

Goat smallholder farmers in the study area generally release their animals in the morning to let them graze freely in the surrounding grazing areas for around 4–6 h during the rainy season (September-March) and 6–8 h in the dry season (May–August). Farmers typically offered supplementary feeding in the morning before releasing their animals to browse and graze. Supplementary feeding with a range of grains, crop residues and cut plants (e.g. maize stover, lablab and cowpea crop residues) was allowed as standard farm practice in both TST and plant-TST groups, but in the latter case farmers were instructed to supply the selected plant species to individual goats specifically in response to health indicators.

Two TST schemes were implemented: TST and plant-TST. In the TST group, no specific supplementation with the selected plants was supplied, but individual goats were treated with an anthelmintic drug when they met set criteria for poor health based on the FPC. On farms assigned to the plant-TST group, the same monitoring and thresholds for anthelmintic treatment were set, but supplementary feeding with bioactive plants was additionally offered to goats in borderline health status. A treatment decision chart was designed to facilitate the application of the interventions for each group of farmers (Fig. 3c). It was hypothesised that goats offered additional plant supplementation would require less anthelmintic treatment as a result of the beneficial effects of those plants on their health, or even considering a synergistic effect between the anthelmintic and the improved nutrition. No non-intervention group was included for ethical reasons.

Farmers were visited twice at the start of the project, two weeks apart, to induct them into the study and provide training, as well as to set up the trial and collect baseline data. Thereafter, data collection visits were scheduled at monthly intervals.

Based on the decision chart (Fig. 3b) and according to FPC scores, plant-TST farmers harvested the plants and fed goats meeting the intervention criteria of ~ 250 g FB/goat every morning for 8–12 days. Goats in either group meeting the criteria for drug intervention were given levamisole 3%m/v + rafoxanide 3% m/v at a dose of 2.5 ml/10 kg body weight (BW) (Eradiworm plus, Afrivet, South Africa). Those goats in the plant-TST group that met the criteria for drug treatment were also offered concurrent plant supplementation. Goats on Plant-TST farms not meeting criteria for drug nor plant intervention were not specifically supplemented with the experimental plants, just the regular basal diet, mainly *Acacia* spp, *Lablab purpureus* and grazing time.

Data collection was limited to adult goats´ FPC scores, age and weight, physiological status, number of kids, and farmer events during each visit. Goat health status was classified according to FPC, and consequently guided the type of intervention (Fig. 3c): No intervention (“Healthy status”): Goats were considered healthy according to good FPC scores. Plant intervention (“Borderline status”): Goats showing limited signs of ill-health associated with parasitism. Drug intervention (“Sick status”): Goats showing unfavourable FPC scores, indicating health issues or “sickness”.

Faecal samples were taken from all adult female goats that required drug and/or plant interventions. Some goats with no intervention were also sampled for comparison within kraals. Samples were taken immediately on voiding into clean sealed plastic bags and kept refrigerated in a cooler box with refrigerants at ≤ 8 °C prior to analysis.

### Feed chemical analyses

Dry matter content was determined at 105 °C until no further loss of weight was recorded. Dried material was then milled to 1 mm and 3 g of the resulting material was furnaced (505 °C for 12 h, ramp rate 2 °C/min) to determine the ash content and by mass difference organic matter. Detergent fibre (expressed inclusive of residual ash) was determined as described in Davies et al. ^72^ with the exception of using oven-dried, not freeze-dried material. Samples were defatted with acid detergent, then starch was transformed to soluble sugars by treating with α-amylase. The soluble material was removed by boiling in neutral solution and the remaining insoluble material was weighed to determine the neutral detergent fibre. From the soluble material, acid detergent fibre was determined using the Ankom 220 analyser (ANKOM Technology Corp., Macedon, NY, USA). Total N content of the forage was determined by the Kjeldahl technique (FOSS Kjeltec 8400 analyser, Foss Co. Ltd, Denmark). Crude protein was calculated from total N content multiplied by 6.25. Total phenols and total tannins were determined by the Folin-Ciocalteu method, and Butanol-HCl method was performed to determine condensed tannins content equivalent to leucocyanidins as standard ^73^.

### Parasitological measurements

GIN egg per gram of faeces (EPG) was estimated using the modified McMaster methodology at a sensitivity of 50 EPG ^74^.

The third quartile (75% of the population) was calculated considering only healthy goats, in an attempt to determine the “normal” FEC threshold during the experimental study. Following the 80:20 rule explaining the overdispersion by 80% of whole eggs, shedding is only by 20% of the animals ^75^. Therefore, it was estimated that at least 75% of the healthy animals from the farmers should have had FECs of 400 EPG.

### Statistical analyses

Temperature, precipitation, and humidity were summarised for descriptive statistics. Inter-farm means of micro temperature and humidity were calculated for each group to visualise their dynamics during the study.

As categorical data non-parametric test was performed comparing the proportions of animal statuses. A two-proportion Z-test was used to compare the proportion of animals with sick or healthy status in the Plant-TST vs TST group.

The proportion of goats assigned different FPC scores was plotted individually using R software, and the TST interventions implemented were shown through descriptive statistics ^76^.

Survival analysis was adapted in order to assess whether plant supplementation prolonged the healthy period until drug treatment was required, i.e., survival-to-treatment. Survival period (days) per goat was thus determined as the difference between the first date of drug intervention and the first day in the study. The number of plant interventions required was also counted for each goat. Resulting survival plots depict the probability of animals staying healthy for a period 1d with or without a specific event (drug intervention) ^77^. Kaplan–Meier analyses were performed between goats in the plant-TST and TST groups. Survival curves were calculated to compare survival-to-treatment between the TST groups, the plant species, and the number of plant interventions received during the study ^76^.

For the generalised linear mixed models, the BW and FEC were considered dependent variables, and the assumptions of normal distribution and homogeneity of variance were explored using Shapiro Wilk’s and Levene’s test, respectively (R Package “ggpubr”). The FPC outcomes (Nasal discharge presence, FAMACHA score, bottle jaw presence, BCS score, Dag score), TST group (TST-Plant or TST), age (years), physiological status (pregnant, lactating or non-lactating), health status (sick, borderline or healthy), time (time points), and plant species (*T. sericea* or *V. rotundifolium*) were classified as independent variables. The health status × TST interaction was included in the models according to the relevant hypothesis.

A generalised linear mixed model with repeated measurements was formulated (Package “GlmmTMB”). The individual identification number of each goat was included as a random factor in the final model and each goat was considered as the experimental unit. The Poisson, Gaussian, Negative binomial, and Zero-Inflated binomial distributions were assessed, and the best distribution was chosen according to the lowest Akaike Information Criterion value ^78,79^. Moreover, pairwise comparisons were performed with the “emmeans package”, using the functions “response” on the significant variables to obtain the log-transformed values and “pairwise” for the means comparisons from the best model ^76,80,81^

Finally, Wilcoxon signed-rank test was used to investigate the effect of deworming of sick goats on their parasite burden, by comparing the FEC pre- and post-drug intervention within Plant-TST and TST groups (“ggpaired package”).

Only significant interactions are shown in the results section, with a *P*-value < 0.05 considered significant. All procedures were performed in R-software version 4.1.3 (2022–03-10) ^76^

## Supplementary Information

Below is the link to the electronic supplementary material.

Supplementary Material 1

## Data Availability

This manuscript does not report data generation or analysis. Data will be made available by the PI (Prof. Eric R. Morgan: Eric.Morgan@qub.ac.uk) upon reasonable request.
