# Supplementary material for: Targeted supplementation with bioactive plants sustainably improves goat health and decreases antiparasitic drug use on smallholder farms
Source: Sci Rep. 2026 Mar 3;16:11805. doi: 10.1038/s41598-025-34862-y (PMC13065785; doi:10.1038/s41598-025-34862-y)
Supplement: Supplementary file 1 — Supplementary Material 1 [file 41598_2025_34862_MOESM1_ESM.docx]

**SUPPLEMENTARY MATERIAL**

**Targeted supplementation with bioactive plants sustainably improves goat health and decreases antiparasitic drug use on smallholder farms**

Honest Machekano^1,2^, Javier Ventura-Cordero^3^, Paul M. Airs^3^, Lovemore C. Gwiriri^4^, Andrew Cooke^4,11^, Joseph Virgil^1^, Andrew Safalaoh^6^, Patson C. Nalivata^6^, Michael R.F. Lee^7^, Taro Takahashi^4,8^, Jan van Wyk^9^, Eric R. Morgan^3^* and Casper Nyamukondiwa^1,10^

^1^Department of Biological Sciences and Biotechnology, Botswana International University of Science and Technology, Palapye, Botswana

^2^Department of Zoology and Entomology, University of Pretoria, Private Bag X20, Hatfield 0028, Pretoria, South Africa

^3^School of Biological Sciences, Queen’s University Belfast, Belfast, Antrim, United Kingdom

^4^Net Zero and Resilient Agriculture, Rothamsted Research, Okehampton, Devon, United Kingdom

^5^Bristol Veterinary School, University of Bristol, Langford, Somerset, United Kingdom

^6^Animal Science Department, Lilongwe University of Agriculture and Natural Resources, Lilongwe, Malawi

^7^School of Sustainable Food and Farming, Harper Adams University, Newport, Shropshire, United Kingdom

^8^Bristol Veterinary School, University of Bristol, Langford, Somerset, United Kingdom

^9^Department of Veterinary Tropical Diseases, University of Pretoria, Pretoria, South Africa

^10^Department of Zoology and Entomology, Rhodes University, Makhanda 6140, South Africa

^11^School of Life Sciences, University of Lincoln, Lincoln, UK

*Corresponding author

E-mail: [honest.machekano@up.ac.za](mailto:honest.machekano@up.ac.za); [Eric.Morgan@qub.ac.uk](mailto:Eric.Morgan@qub.ac.uk)

**Table S1. Effect of plant-based treatment in the goats’ faecal egg counts applying the Plant-Targeted Selective Treatment and Drug-Targeted Selective Treatment.** ^a-d^ Different literals in the same column indicate significant differences P<0.05.

| Factor | Number of events | Level | Mean (EPG) | LCL | UCL |
| --- | --- | --- | --- | --- | --- |
| FAMACHA | 124 | 1 | 238 ^ab^ | 143 | 397 |
|  | 735 | 2 | 271 ^ab^ | 177 | 414 |
|  | 1363 | 3 | 293 ^a^ | 194 | 443 |
|  | 401 | 4 | 408 ^b^ | 258 | 644 |
|  | 24 | 5 | 359 ^ab^ | 138 | 932 |
| Body Condition Score | 2 | 0.5 | 150 ^ab^ | 23 | 982 |
|  | 213 | 1 | 528 ^ab^ | 375 | 743 |
|  | 1078 | 1.5 | 536 ^a^ | 427 | 672 |
|  | 1016 | 2 | 416 ^b^ | 331 | 522 |
|  | 301 | 2.5 | 416 ^ab^ | 314 | 553 |
|  | 50 | 3 | 323 ^ab^ | 203 | 516 |
|  | 1 | 3.5 | 111 ^ab^ | 16 | 776 |
| Physiological status | 718 | Lactating | 348 ^a^ | 223 | 543 |
|  | 738 | Pregnant | 313 ^ab^ | 200 | 490 |
|  | 1055 | Not lactating | 268 ^b^ | 171 | 420 |
| Timepoints | 102 | Jan-20 | 501 ^abc^ | 300 | 300.4 |
|  | 146 | Feb-20 | 629 ^a^ | 386 | 386.4 |
|  | 93 | Mar-20 | 545 ^ab^ | 323 | 323.1 |
|  | 176 | May-20 | 276 ^cd^ | 172 | 172.2 |
|  | 297 | Jun-20 | 287 ^cd^ | 180 | 180 |
|  | 251 | Jul-20 | 128 ^e^ | 79 | 78.7 |
|  | 238 | Aug-20 | 229 ^d^ | 140 | 140.4 |
|  | 193 | Sep-20 | 190 ^de^ | 117 | 116.9 |
|  | 143 | Oct-20 | 327 ^bcd^ | 201 | 200.5 |
|  | 261 | Nov-20 | 436 ^abc^ | 274 | 273.8 |
|  | 96 | Dec-20 | 302 ^abcde^ | 142 | 141.5 |
|  | 199 | Jan-21 | 519 ^ab^ | 320 | 320.2 |
|  | 210 | Feb-21 | 243 ^d^ | 149 | 149 |
|  | 87 | Mar-21 | 225 ^de^ | 133 | 132.7 |
|  | 93 | Apr-21 | 206 ^de^ | 121 | 120.8 |
|  | 86 | May-21 | 340 ^abcd^ | 203 | 203 |
| Plant species | 602 | *Viscum rotundifolium* | 374 ^a^ | 236 | 592 |
|  | 418 | No plant | 327 ^ab^ | 204 | 523 |
|  | 634 | *Terminalia sericea* | 238 ^b^ | 151 | 375 |

**Table S2. Effect of plant-based treatment in the goats’ body weight applying the Plant-Targeted Selective Treatment and Drug-Targeted Selective Treatment.** ^a-i^ Different literals in the same column indicate significant differences P<0.05.

| Factor | Number of events | Level | BW Mean (kg) | LCL | UCL |
| --- | --- | --- | --- | --- | --- |
| Nasal | 5278 | 0 | 33.9 ^a^ | 31.8 | 35.9 |
|  | 2609 | 1 | 33.4 ^b^ | 31.3 | 35.5 |
|  | 1 | 2 | 29.1 ^ab^ | 20.1 | 38.2 |
| FAMACHA | 460 | 1 | 31.2 ^a^ | 27.7 | 34.8 |
|  | 3838 | 2 | 31.9 ^b^ | 28.3 | 35.5 |
|  | 2983 | 3 | 32.4 ^bc^ | 28.8 | 35.9 |
|  | 562 | 4 | 33.3 ^c^ | 29.5 | 37.0 |
|  | 36 | 5 | 31.9 ^abc^ | 28.0 | 35.9 |
| Submandibular oedema | 21 | Yes | 30.5 ^a^ | 27.1 | 34.0 |
|  | 7877 | No | 33.7 ^b^ | 29.7 | 37.7 |
| Body Condition Score | 4 | 0.5 | 28.4 ^abcde^ | 22.7 | 34.0 |
|  | 292 | 1 | 28.9 ^a^ | 25.2 | 32.6 |
|  | 2399 | 1.5 | 30.2 ^b^ | 26.7 | 33.8 |
|  | 3340 | 2 | 32.0 ^c^ | 28.4 | 35.5 |
|  | 1467 | 2.5 | 33.4 ^d^ | 29.9 | 37.0 |
|  | 358 | 3 | 34.5 ^ef^ | 30.9 | 38.1 |
|  | 21 | 3.5 | 37.5 ^e^ | 33.5 | 41.5 |
| Dag Score | 7549 | 0 | 34.7 ^a^ | 31.5 | 37.9 |
|  | 249 | 1 | 34.0 ^ab^ | 30.8 | 37.3 |
|  | 52 | 2 | 32.2 ^b^ | 28.8 | 35.7 |
|  | 24 | 3 | 32.6 ^ab^ | 28.9 | 36.3 |
|  | 6 | 4 | 33.0 ^ab^ | 28.2 | 37.8 |
|  | 1 | 5 | 26.3^ab^ | 17.0 | 35.5 |
| Timepoint | 357 | Jan-20 | 33.1 ^bcd^ | 29.4 | 36.7 |
|  | 406 | Feb-20 | 33.2 ^bc^ | 29.6 | 36.8 |
|  | 948 | Mar-20 | 33.6 ^b^ | 30.0 | 37.2 |
|  | 390 | May-20 | 34.7 ^a^ | 31.1 | 38.4 |
|  | 718 | Jun-20 | 33.6 ^b^ | 30.0 | 37.2 |
|  | 581 | Jul-20 | 33.2 ^bc^ | 29.6 | 36.9 |
|  | 594 | Aug-20 | 32.0 ^def^ | 28.4 | 35.7 |
|  | 393 | Sep-20 | 29.4 ^i^ | 25.8 | 33.0 |
|  | 315 | Oct-20 | 30.1 ^hi^ | 26.4 | 33.7 |
|  | 585 | Nov-20 | 31.1 ^gh^ | 27.4 | 34.7 |
|  | 248 | Dec-20 | 31.6 ^efg^ | 28.0 | 35.3 |
|  | 459 | Jan-21 | 31.7 ^efg^ | 28.1 | 35.3 |
|  | 482 | Feb-21 | 31.3 ^fg^ | 27.7 | 34.9 |
|  | 482 | Mar-21 | 31.0 ^gh^ | 27.4 | 34.7 |
|  | 473 | Apr-21 | 32.0 ^efg^ | 28.3 | 35.6 |
|  | 452 | May-21 | 32.5 ^cde^ | 28.9 | 36.1 |
| TST | 1895 | Borderline | 31.5 ^a^ | 27.8 | 35.2 |
|  | 1216 | Healthy | 31.0 ^a^ | 27.3 | 34.6 |
|  | 458 | Sick | 31.9 ^a^ | 28.3 | 35.4 |
| Plant-TST | 1777 | Borderline | 33.0 ^b^ | 29.4 | 36.7 |
|  | 2193 | Healthy | 32.9 ^b^ | 29.2 | 36.6 |
|  | 344 | Sick | 32.6 ^b^ | 29.0 | 36.1 |


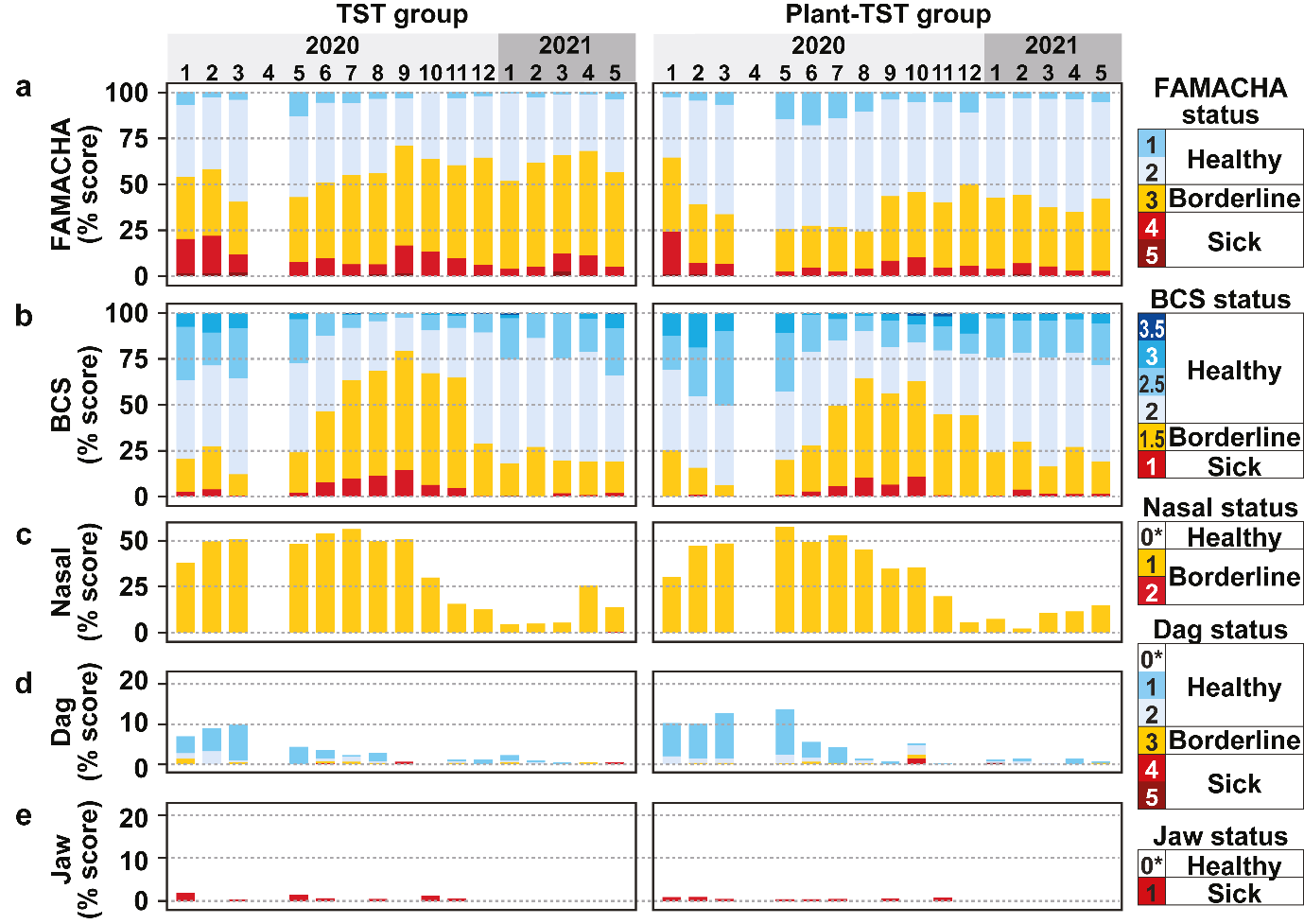


**Figure S1**. Five Point Check© scores of all goats per TST group over the study period. The monthly total measurements of all goats from TST and plant-TST groups for (a) FAMACHA eye scores, (b) body condition scores, (c) nasal discharge scores, (d) dag / scour scores, (e) bottle jaw / pitting oedema scores. The month of the year is represented by a number on the year in the X-axis, where 1=Jan up to where 12=Dec. Data was not collected during April 2020 due to COVID-19 lockdown restrictions.


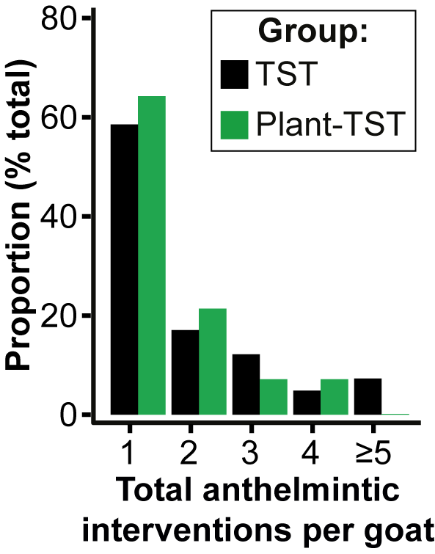


**Figure S2**: Total anthelmintic interventions provided for TST and Plant-TST regimes.


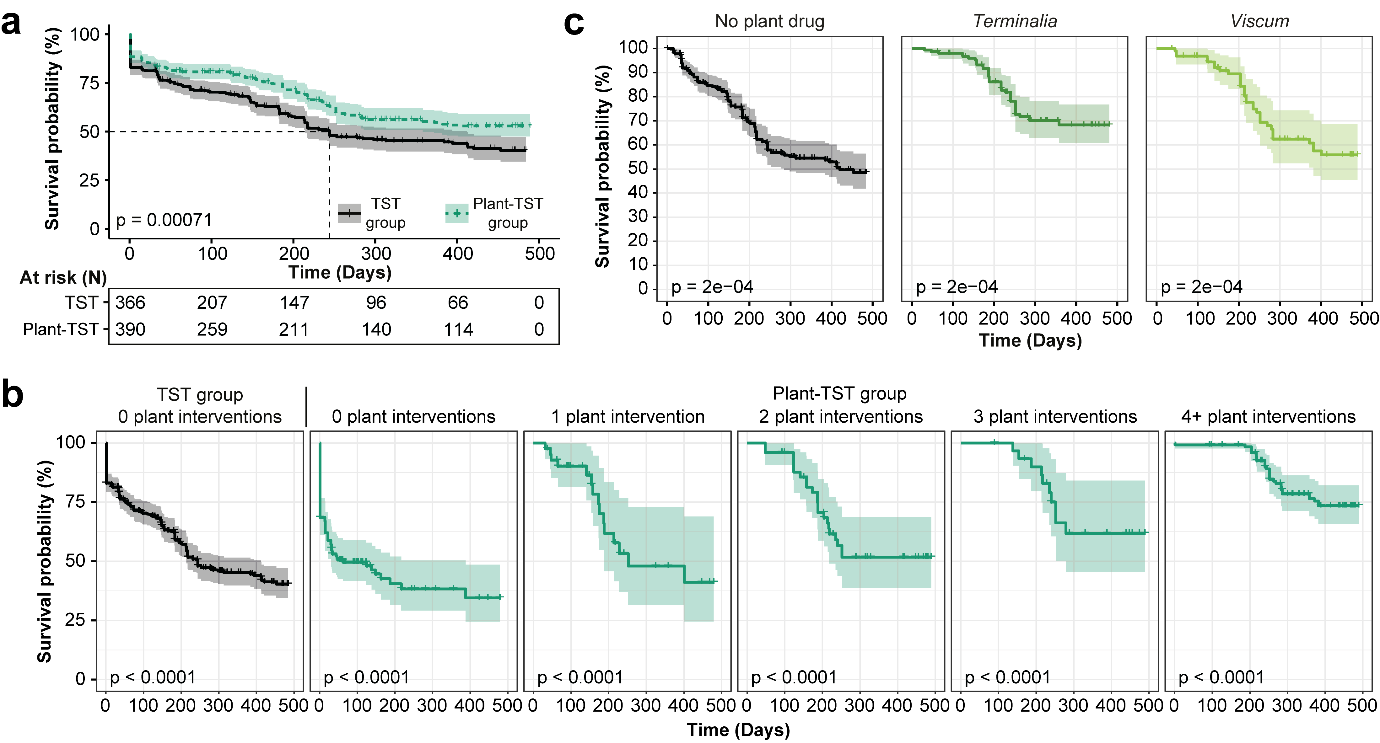


**Figure S3.** Kaplan-Meier survival curve analysis of anthelmintic intervention events. (a) TST interventions reduced the number of goats at risk of anthelmintic drug intervention over time. (b) Plant interventions decreased the likelihood for future drug intervention need. (c) Plant supplementations differed in their impact on goat health over time.


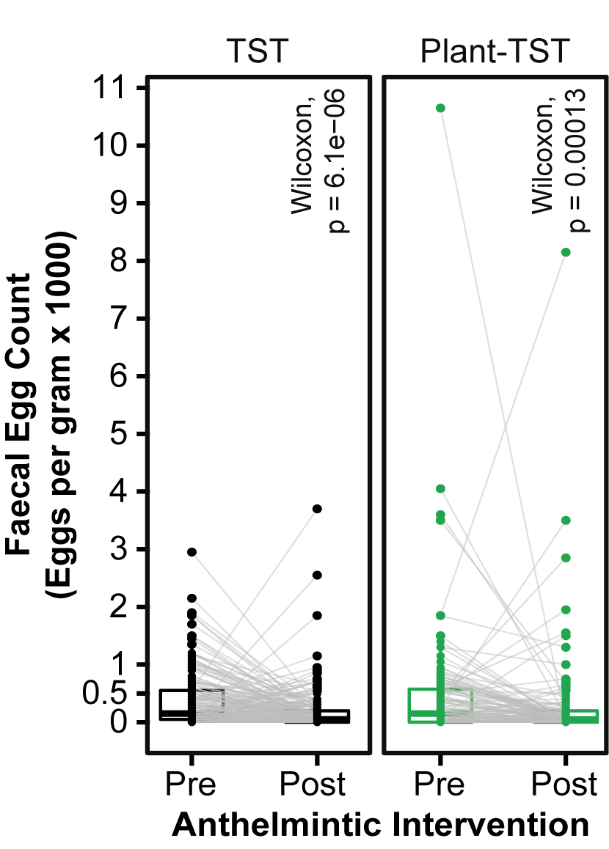


**Figure S4**. Total faecal worm egg count as measured by McMasters pre-anthelmintic and two weeks post-anthelmintic intervention for both TST regimes.
